# Improved characterisation of coral-associated fungal communities using host DNA depletion and a novel ITS primer

**DOI:** 10.1093/ismeco/ycag060

**Published:** 2026-03-13

**Authors:** Ming Sheng Ng, Min Yi Chin, Golam Rabbani, Lynn Drescher, Benjamin J Wainwright, Ying Chang

**Affiliations:** Department of Biological Sciences, National University of Singapore, 16 Science Drive 4, Singapore 117558, Singapore; Department of Biological Sciences, National University of Singapore, 16 Science Drive 4, Singapore 117558, Singapore; Department of Biological Sciences, National University of Singapore, 16 Science Drive 4, Singapore 117558, Singapore; Department of Biological Sciences, National University of Singapore, 16 Science Drive 4, Singapore 117558, Singapore; Department of Biological Sciences, National University of Singapore, 16 Science Drive 4, Singapore 117558, Singapore; Department of Biological Sciences, National University of Singapore, 16 Science Drive 4, Singapore 117558, Singapore

**Keywords:** coral mycobiome, marine fungi, host suppression primers, host DNA depletion, fungal metabarcoding

## Abstract

Fungi are increasingly recognised as integral components of the coral holobiont, with predicted roles in nitrogen cycling, nutrient acquisition and promoting stress tolerance. However, characterising coral-associated fungal communities using metabarcoding remains challenging due to extensive co-amplification of host DNA, often leading to low fungal read recovery and an underestimation of diversity. Here, we developed and validated a robust fungal metabarcoding protocol that consistently improved fungal reads across coral species for comprehensive diversity analyses. We addressed host co-amplification through two approaches: (i) host depletion with the Zymo HostZERO Microbial DNA kit and (ii) a newly designed forward primer, ITS3-CoralF, with mismatches to coral DNA to reduce their amplification when paired with the ITS4 reverse primer. We assessed the extraction kit and primer biases and performance across reef sediment samples, and the tissue and skeletal compartments of three species of corals, *Pocillopora acuta, Pachyseris speciosa*, and *Diploastrea heliopora*. The HostZERO kit consistently reduced non-target eukaryotic DNA across sample types, enhancing the detection of rare fungi despite shifts in community composition. In coral tissues, ITS3-CoralF achieved at least an 800-fold increase in fungal reads compared to universal primers, though with some bias against rare taxa. For skeletons, thorough removal of tissue prior to total DNA extraction was sufficient, though ITS3-CoralF further improved fungal recovery. Together, our results provide a validated pipeline for profiling fungi across coral hosts, allowing improved recovery of fungal diversity and a deeper understanding of the coral mycobiome.

## Introduction

Tropical coral reefs are among the most diverse and productive ecosystems on Earth, supporting between three and six million species of marine organisms [1, 2]. This remarkable ecological success relies heavily on the complex symbiosis between coral hosts and microorganisms across all domains of life within the holobiont [3]. In particular, *Symbiodiniaceae* endosymbionts supply up to 90% of the coral’s nutritional requirements through photosynthate translocation [4, 5], while also contributing to skeletal calcification [6, 7] and nutrient recycling [8]. Corals also harbour a diverse assemblage of bacteria that produce essential amino acids and secondary metabolites crucial for host defence and resilience [9]. Yet, despite the growing recognition of viruses, archaea, and microeukaryotes as integral holobiont members, the roles of these other coral associates remain poorly understood, among which fungi remain particularly enigmatic.

Fungi are found in nearly every marine habitat ever explored to date, from nearshore coastal mangrove and seagrass sediments [10, 11] to deep-sea extreme environments and the igneous oceanic crust [12, 13], where they play key roles especially in decomposition and cross-domain interactions [14, 15]. Many marine fungi form close associations, sometimes mutualistic, with a wide range of marine hosts, including macroalgae [16], sponges [17], and nudibranchs [18], although the nature and function of these associations remain poorly understood. In scleractinian corals, fungi were initially regarded as pathogens due to their invasion of carbonate skeletons and antagonistic attacks towards the endolithic *Oestrobium* algae, and in some cases, even the coral polyps themselves [19, 20]. Today, fungi are generally recognised as important components of a healthy coral holobiont [21], and are readily recovered across coral species [22, 23]. Metagenomic analysis further revealed that coral-associated fungi harbour many diverse genes involved in nitrate/nitrite reduction and ammonia assimilation pathways, suggesting a potential role in nitrogen cycling within the holobiont [24–26].

Despite the increasing recognition of their importance within the coral holobiont, it remains challenging to characterise coral-associated fungi via metabarcoding sequencing due to the overwhelming co-amplification of host and other off-target eukaryotic DNA. This is because universal fungal primers mainly target the highly conserved eukaryotic 18S, 28S, and 5.8S regions, which show high homology between coral and fungi [27]. Coupled with low fungal biomass, this often results in extremely low fungal sequencing coverage with typically only 0.2%–2% reads assigned to the fungal kingdom, and thus a severe underestimation of their diversity [28–30]. Mismatch primers provide a promising solution, especially mismatches at the 3′ end that disproportionately reduce priming efficiency, with multiple or consecutive mismatches often entirely inhibiting amplification [31, 32]. A classic example is the bacterial 799F primer, which preferentially amplifies bacterial 16S rRNA genes while avoiding host chloroplast sequences with just two mismatches at the 3′ end [33]. Instead of 3′ mismatches, previous attempts to target fungal sequences through new primer designs [34] or fungal phylum-specific primers [35] largely relied on sporadic base differences across the primer-template region, which proved insufficient to suppress coral DNA amplification and recover fungal diversity. Newly designed mismatch primers should thus deliberately exploit primer-template mismatches on the 3′ end to suppress host amplification. Compared to other host-DNA suppression methods like blocking primers or peptide nucleic acid (PNA) clamps, mismatch primers are also more cost-effective, and can be easily incorporated into existing metabarcoding workflows to facilitate the discovery and our understanding of coral-associated fungi.

Another promising approach to reduce host co-amplification and enhance microbial signals are through Host DNA depletion kits. These kits selectively lyse host cells, enzymatically degrade their DNA, and preserve more robust microbial cells prior to extraction [27]. Using this strategy, Rabbani et al. achieved an average of 76% fungal reads from *Pocillopora acuta* using the HostZERO Microbial DNA kit (Zymo Research, CA, USA) with standard fungal primers [36]. While this kit has successfully enriched microbial signals across marine bivalves and human samples [37, 38], it has only been applied to one coral species, and remains to be tested across multiple species. Without a reliable approach to recover fungal sequences across diverse coral hosts, our ability to better understand coral-fungal associations is limited. In fact, because standard metabarcoding workflows cannot consistently detect fungal signals, it remains challenging to determine whether fungi are truly coral-associated and confer benefits to their host, or simply occur through random events, especially during feeding [27, 39].

While there has been previous work on coral-mycobiome characterisation, the work presented here was motivated by the inconsistency of current published methodologies in gathering fungal reads. In our own tests with four primer pairs (ITS1F/ITS2, ITS5/5.8S_Fungi, fITS7/ITS4, and ITS86F/ITS4) that were previously used to characterise fungal communities in corals and other marine systems [10, 40–43], 99% of all sequencing reads were lost to coral ITS sequences, leaving our data unusable (unpublished dataset). As such, in this study, we aim to improve the characterisation of coral-associated mycobiome through the HostZERO Microbial DNA kit and a newly designed mismatched primer, ITS3-CoralF. We evaluated the performance of these approaches on tissue and skeletal samples of three coral species: *Pocillopora acuta, Pachyseris speciosa*, and *Diploastrea heliopora*. We also assessed potential taxonomic biases using reef sediments as non-coral controls. Our findings provide practical recommendations for robust coral mycobiome profiling and aim to catalyse broader efforts to uncover the full diversity of coral-associated fungi.

## Materials and methods

### Sample collection

Three sediment samples were collected from each of three reef sites in Singapore, Pulau Jong (Latitude: 1.215461, Longitude: 103.786729), Pulau Hantu (Latitude: 1.226276, Longitude: 103.752899), and Lazarus Island (Latitude: 1.229914, Longitude: 103.848487) in January 2024 (n = 9). Samples were collected by SCUBA with a 50 ml falcon tube, kept shaded on ice during transport, then centrifuged at 11 000 × *g* for 12 min at 4° C to replace seawater with 100% ethanol for preservation in −80° C.

For coral samples, five fragments (~5 cm^2^) were collected from *D. heliopora, P. speciosa*, and *Pocillopora acuta*, using SCUBA from Pulau Jong (n = 15). These three coral species represent three major growth forms: sub-massive/encrusting, laminar, and branching, from three different Scleractinia families: Diploastreidae, Agariciidae, and Pocilloporidae, providing a broad assessment of the protocol’s applicability. Fragments were transported in shaded, cooled seawater and processed immediately upon return. Coral tissues were first separated from skeletons by air-brushing with autoclaved seawater [44]. After tissue had been stripped, the remaining skeletons were briefly rinsed with 70% ethanol and further air-brushed with autoclaved seawater to thoroughly remove residual coral tissues. Skeletons were then lightly crushed using autoclaved mortars and pestles. Both the tissue slurry and grounded skeletal samples were centrifuged at 11 000 × *g* for 12 mins at 4° C to be preserved in 100% ethanol in −80 °C.

### DNA extraction

To assess the taxonomic and compositional biases introduced by DNA extraction methods, the DNeasy PowerLyzer PowerSoil Kit (Qiagen, Germany) and the HostZERO Microbial DNA Kit (Zymo Research, CA, USA) were used to extract DNA from sediment samples. The PowerSoil kit is an effective total DNA extraction kit widely used in many standardised metabarcoding protocols across various sample types [45]. Whereas the HostZERO kit first selectively lyses metazoan cells, allowing the subsequent depletion of non-target DNA, this technique keeps the more robust microbial cells intact for further DNA extraction and purification. This effectively enriches microbial DNA, allowing for more accurate and comprehensive community characterisation [37]. Detailed protocols, including modifications, are outlined in the Supplementary Methods.

Both kits were subsequently applied to coral tissue pellets. For coral skeleton samples, only the PowerSoil kit was used, as prior tissue removal was expected to minimise host contamination for sufficient fungal reads recovery.

### Primer design, DNA amplification, and sequencing

**Figure 1:**
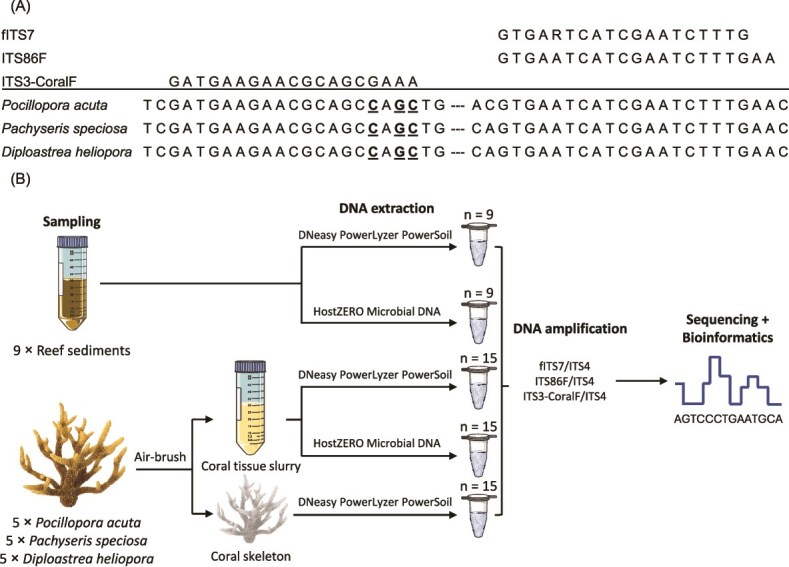
(A) Alignment of the three forward primers, fITS7, ITS86F, and ITS3-CoralF, against the conserved 5.8S sequences of the three coral species in this study. Bolded and underlined bases indicate the designed mismatches between the ITS3-CoralF and the coral template, while the dashes represent 26 bp of identical sequence that was omitted between priming sites. (B) Schematic overview of workflow across the three sample types, sediments, coral tissue, and skeleton, in this study.

To reduce coral host amplification, we designed a new forward primer (ITS3-CoralF; 5′- GAT GAA GAA CGC AGC GAA A − 3′) targeting the conserved 5.8S region to amplify the ITS2 nuclear DNA region when paired with the universal reverse primer ITS4 (5′- TCC TCC GCT TAT TGA TAT GC -3′) [46]. This primer shifts the ITS3 primer four bases towards the 3′ end to introduce two to four 3′ mismatches to coral DNA (Fig. 1A, Fig. S1). The ITS2 region was selected for its higher sequence variability, allowing finer taxonomic resolution and greater richness recovery [47, 48]. Complete details on primer design are provided in the Supplementary Methods.

Fungal specificity was evaluated via in-silico PCR against 93 085 full-length fungal ITS sequences downloaded from the UNITE database (v10.0) using PrimerEvalPy [49], comparing ITS3-CoralF against two common fungal universal forward primers, fITS7 (5′- GTG ART CAT CGA ATC TTT G -3′) [50] and ITS86F (5′- GTG AAT CAT CGA ATC TTT GAA -3′) [51]. Off-target amplification was assessed against 604 Anthozoan sequences taken from the UNITE eukaryote database (v10.0), including 389 representative Scleractinia sequences and nine other orders such as Alcyonacea and Zoantharia. To better simulate real PCR conditions, 0 to 2 mismatches were allowed between primer-template pair, except for the final two 3′ bases, which were excluded from mismatch tolerance using a custom R script.

To evaluate diversity and compositional biases of ITS3-CoralF and the HostZERO kit, in-vitro PCR was conducted with the three forward primers paired with ITS4 on sediment samples extracted with both DNA extraction kits. These three primer sets were also used on coral tissue and skeleton samples to determine whether ITS3-CoralF can reduce host co-amplification to improve fungal read coverage (Fig. 1B).

Detailed PCR cycling conditions are provided in the Supplementary Methods. Negative extraction, PCR controls and the Mycobiome Genomic DNA Mix mock community were included to detect potential contamination and to evaluate amplification success. All 204 samples (189 samples +3 mock +6 blanks, Fig. 1B) were sequenced using the Illumina MiSeq platform across three runs with a 30% PhiX spike by Macrogen, Inc (600 cycles, V3 chemistry, 300 bp paired-end reads).

### Bioinformatics and statistical analysis

To account for amplicon length variability and intragenomic variation within the ITS region, sequences were clustered into 97% operational taxonomic units (OTUs), with amplicon sequence variant (ASV)-based analyses conducted in parallel as cross-validation [52, 53]. Bioinformatics analysis was performed using PipeCraft 2.0 (v1.1.0) [54], encompassing quality trimming, paired-end read assembly, chimera filtering with VSEARCH (v2.29.4) [55]. The ITS2 regions were then extracted from chimera-filtered reads using ITSx (v1.1.3) [56] before clustering. To ensure robust analyses, singletons and doubletons were also removed prior to downstream steps. Analyses based on ASVs produced qualitatively similar patterns to those obtained using 97% OTUs, indicating that the main conclusions were robust to bioinformatics strategy. All results reported below pertain to OTU-based analysis, for results of the ASV-based analysis see Supplementary Information section 2 (Figs S6–S8).

Taxonomy was assigned with QIIME2’s *q2-feature-classifier* function (v2024.10.0) [57]. To improve taxonomic assignments, unassigned or Kingdom-level OTUs were reannotated using *megablast* (v2.16.0) against the NCBI nucleotide database [58], following recommended e-value thresholds [52]. Rarefaction curves were generated to determine whether fungal read depths were sufficient to recover all diversity. Subsampling with 999 iterations was then performed to account for uneven sequencing depth across samples [59].

All downstream statistical analyses were performed in R. For sediment samples, analysis of variance (ANOVA) was used to determine if alpha-diversity indices were significantly affected by DNA extraction methods, primer choice, and their interaction effects. Site was included as a covariate to account for spatial environmental heterogeneity, while testing for consistent methodological effects across samples. Significant effects were followed up with post-hoc pairwise comparisons with Tukey’s correction (*emmeans,* v1.10.6) [60].

Beta-diversity was assessed using Bray–Curtis dissimilarity and visualised with non-metric multidimensional scaling (NMDS). Homogeneity of dispersions was first checked with *betadisper* (*vegan* package, v2.6.8) [61], before conducting permutational multivariate analysis of variance (PERMANOVA) to investigate if fungal compositions were significantly affected by DNA extraction method, primer choice and their interaction, and sampling site. Significant pairs were further identified with *pairwise.adonis* with Bonferroni’s correction (v0.4.1) [62]. Differentially abundant fungal families across DNA extraction methods and primer pairs were identified using the ANCOM-BC2 framework (v2.4.0) [63].

For coral tissue and skeleton samples, we determined whether library construction methods significantly affected (i) fungal reads percentage, (ii) alpha-diversity indices, and (iii) community compositions. Fungal read percentages of both coral tissue and skeleton samples were log-transformed prior to ANOVA to better meet assumptions of normality and homoscedasticity. Coral tissue libraries generated with the PowerSoil Kit and fITS7/ITS4 or ITS86F/ITS4 primers did not produce sufficient fungal reads and were excluded from downstream analyses.

The effects of library construction method, coral species, and their interaction on Shannon diversity, richness, and evenness were assessed using ANOVA. Fungal richness were log-transformed for both coral tissue and skeleton samples to better meet ANOVA assumptions. Beta-diversity analyses using NMDS and PERMANOVA with Bray–Curtis dissimilarity were then assessed to determine if the same factors significantly affected community composition. Homogeneity of dispersions was checked with *betadisper*. To evaluate overlap across library construction methods, ANOVA was conducted to investigate whether relative abundances of shared fungal families were significantly different across library construction methods.

## Results

### Evaluating the new forward primer ITS3-CoralF with in-silico PCR and mock community

The in-silico PCR indicates that the fungal universal forward primers fITS7 and ITS86F had broader overall coverage to fungal sequences in the UNITE database in comparison to the newly designed ITS3-CoralF. At a tolerance of two mismatches, fITS7 and ITS86F amplified 92.80% and 92.21% of fungal sequences respectively, while ITS3-CoralF amplified 83.97% (Table 1). Despite its lower overall coverage, ITS3-CoralF retained comparable coverage for Ascomycota and Mortierellomycota, and outperformed the other primers in uncommon marine phyla such as Blastocladiomycota and Neocallimastigomycota (Table S1). However, it showed slightly reduced specificity toward Basidiomycota and Chytridiomycota, and complete exclusion of Basidiobolomycota and Kickxellomycota. All three primer pairs successfully amplified all 10 fungal species in the Mycobiome Genomic DNA Mix mock community (Fig. S2), confirming that the ITS3-CoralF primer retains broad fungal coverage.

**Table 1:** Percentage of (A) fungal and (B) Anthozoan sequences amplified by the three forward primers, with 0, 1, and 2 mismatches allowed between primer-template.

	(A) Percentage of fungal sequences amplified (%)			(B) Percentage of Anthozoan sequences amplified (%)		
	0 mismatch	1 mismatch	2 mismatches	0 mismatch	1 mismatch	2 mismatches
ITS3-CoralF	79.05	83.21	83.97	0	0	0
fITS7	79.68	89.81	92.80	67.22	84.10	84.44
ITS86F	75.06	88.68	92.21	66.88	83.77	84.11

Crucially, ITS3-CoralF showed complete exclusion of the 604 Anthozoan sequences, even with two mismatches. In contrast, fITS7 and ITS86F amplified 84.44% and 84.11% respectively (Table 1). Specifically, fITS7 amplified 76.61% of Scleractinia sequences, while ITS86F amplified 76.35% (Table S2). Both primers also amplified 100% of sequences from seven other Anthozoan orders, including Helioporacea, Actiniaria, and Corallimorpharia.

### Assessing primer and DNA extraction biases with reef sediments

To assess primer and extraction biases without coral host interference, we applied the two DNA extraction methods and three primer pairs to reef sediments collected from three sites. Primer choice did not significantly influence sediment fungal Shannon diversity and richness (*P* < .05; Table 2, Table S3). Samples extracted with PowerSoil had similar alpha-diversity indices across primers, with a mean Shannon diversity index of 3.17 ± 0.13 (mean ± SE), OTU richness of 79.30 ± 11.80, and Pielou’s evenness of 0.761 ± 0.019. On the other hand, HostZERO markedly improved fungal detection across all primers, where richness more than doubled to 208 ± 18, while community evenness decreased to 0.669 ± 0.031, resulting in similar Shannon diversity.

**Table 2:** ANOVA was conducted to investigate if primer selection, DNA extraction method, and their interaction effects significantly affected the Shannon diversity, richness, and community evenness of fungal communities associated with reef sediments.

		Df	Sum Sq	Mean Sq	F value	*P*-value
Shannon	Primer	2	160.42	80.209	3.151	.0522
	DNA extraction	1	87.26	87.262	3.428	.0705
	Site	2	78.58	39.289	1.543	.2245
	Primer: DNA extraction	2	154.45	77.226	3.034	.0579
	Residuals	46	1171.04	25.457		
Richness	Primer	2	20 477	10 239	2.396	.1024
	DNA extraction	1	223 108	223 108	52.201	<.0001
	Site	2	107 600	53 800	12.588	<.0001
	Primer: DNA extraction	2	1528	764	0.179	.8368
	Residuals	46	196 603	4274		
Evenness	Primer	2	0.082	0.041	2.936	.0631
	DNA extraction	1	0.112	0.112	7.994	.0069
	Site	2	0.105	0.053	3.759	.0307
	Primer: DNA extraction	2	0.086	0.043	3.059	.0566
	Residuals	46	0.645	0.014		

**Figure 2:**
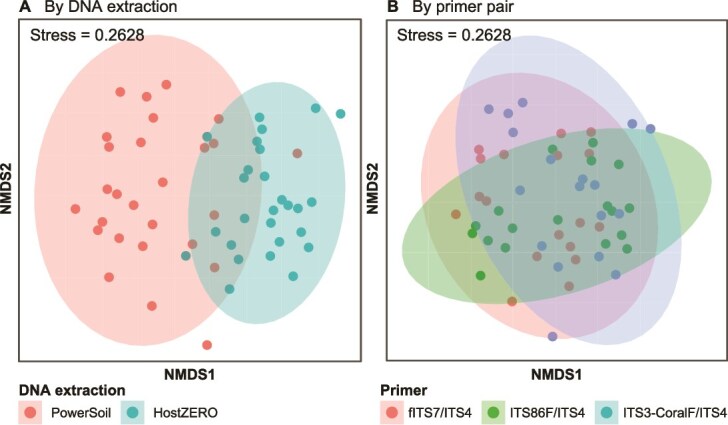
Non-metric multidimensional scaling (NMDS) plot with Bray–Curtis dissimilarities of fungal communities associated with reef sediments, coloured (A) by DNA extraction and (B) by primer pair.

Non-metric multidimensional scaling (NMDS) with Bray–Curtis dissimilarities revealed clear separation of fungal communities by extraction methods, but substantial overlaps by primer sets (Fig. 2), indicating that DNA extraction had a more pronounced effect on fungal composition than primer choice. This is confirmed with permutational analysis of variance (PERMANOVA), with extraction method explaining greater variance (R^2^ = 0.06620, *P =* .001; Table S4) than primer choice (R^2^ = 0.04245, *P* = .033). Group dispersions also differed significantly, with PowerSoil-extracted communities showing greater variability (*P* = .0040; Table S5).

Apart from site effects, interaction effects between DNA extraction and primer choice also significantly affected fungal compositions, indicating marked method-specific biases (R^2^ = 0.04847, *P =* .003). Specifically, fungal compositions differed across primer pairs when paired with the PowerSoil kit, but were similar across HostZERO-extracted communities (Table S6).

**Figure 3:**
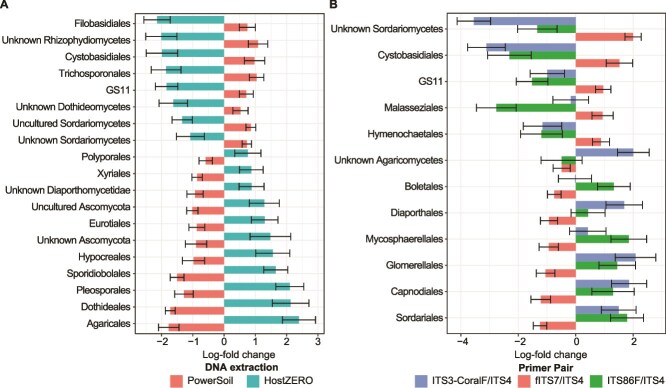
Differentially abundant fungal orders between sediment-associated fungal communities of different (A) DNA extraction methods and (B) primer pairs.

ANCOM-BC2 identified 19 differentially abundant orders between communities of different extraction methods (Fig. 3). The PowerSoil kit captured more unclassified taxa across Ascomycota, Basidiomycota, and Chytridiomycota, while the HostZERO kit captured a greater proportion of Ascomycota (Fig. S3). Primer effects were weaker, with ITS3-CoralF/ITS4 and ITS86F/ITS4 being largely similar with only three differentially abundant taxa. Instead, fITS7/ITS4 had the most differentially abundant taxa compared to the other two primers at 10.

### Both the HostZERO kit and ITS3-CoralF/ITS4 reduced host co-amplification in coral tissue samples for in-depth fungal community characterisation

**Figure 4:**
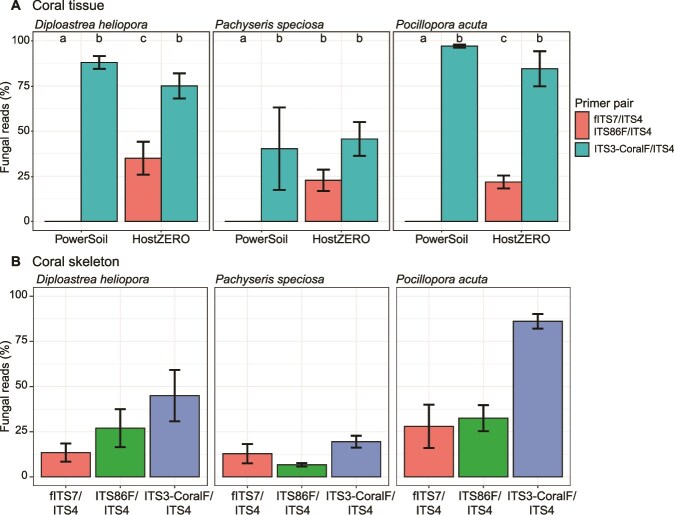
Differences in percentage fungal reads across different coral species, and DNA extraction methods and/or primer pairs in (A) coral tissue and (B) coral skeleton samples. Letters indicate statistically significant differences within each coral species, while error bars indicate standard error. Universal fungal primer pairs fITS7/ITS4 and ITS86F/ITS4 are represented together in coral tissue samples for clarity of presentation.

Metabarcoding library construction typically pairs total DNA extraction with universal fungal primers. However, coral tissue samples extracted with PowerSoil kit and amplified with fITS7/ITS4 or ITS86F/ITS4 yielded negligible reads (0.04%–0.05%) fungal reads (Fig. 4, Tables S7 and S8), failing to generate sufficient data across all samples. In contrast, using the same primers on HostZERO-extracted DNA significantly increased percentage fungal reads to 25% − 28% (*P* = 2.19E^−23^; Table S8), representing a consistent 500–700-fold improvement across the three coral species tested.

**Figure 5:**
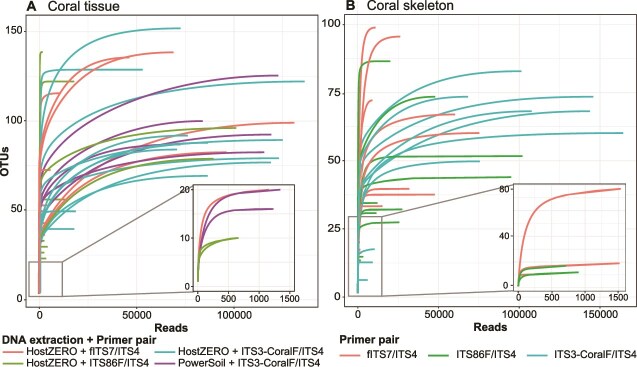
Rarefaction curves indicate that sufficient fungal reads were gathered to reach species saturation in (A) coral tissue and (B) coral skeleton samples, regardless of DNA extraction and primer choice. All skeleton samples were extracted with the PowerSoil kit. The insets represent the bottom five samples with the least number of fungal reads.

Using ITS3-CoralF/ITS4 on DNA extracted with the PowerSoil kit also greatly improved fungal reads percentage (*P* = 1.98E^−9^), although this effect was slightly coral-species specific (*P* = .0458). Specifically, ITS3-CoralF/ITS4 performed best on *Pocillopora acuta*, increasing fungal reads to 97.10% ± 0.84%, followed by *D. heliopora* (88.00% ± 3.55%) and *P. speciosa* (40.30% ± 22.80%). Overall, the combination of PowerSoil kit with ITS3-CoralF/ITS4 produced the most fungal reads at 75.10% ± 9.76% across all coral species. Across retained samples with sufficient sampling depth, 575 OTUs were recovered at 52556 ± 5914 fungal reads per sample, with a minimum of 631 reads used as the rarefaction threshold (Fig. 5A).

Given method-specific biases in fungal diversity and community composition, library construction method was treated as a single factor in subsequent analyses. While construction methods significantly affected Shannon diversity, it was only slightly lower in communities extracted with PowerSoil with ITS3-CoralF/ITS4 at 1.64 ± 0.16 compared to those with HostZERO and fITS7/ITS4 at 2.25 ± 0.12. This was largely driven by the lower evenness in PowerSoil and ITS3-CoralF/ITS4 communities at 0.456 ± 0.056 relative to HostZERO and fITS7/ITS4 communities at 0.686 ± 0.034 (*P* = .0311, Table S9). In contrast, fungal richness were similar across all four library construction methods at 51.27 ± 4.54 (Table S10). Although significant interaction effects were observed between library construction methods and coral species (*P* < .05), they were considered minor as no method consistently affected alpha-diversity indices for any coral species (Table S11).

**Figure 6:**
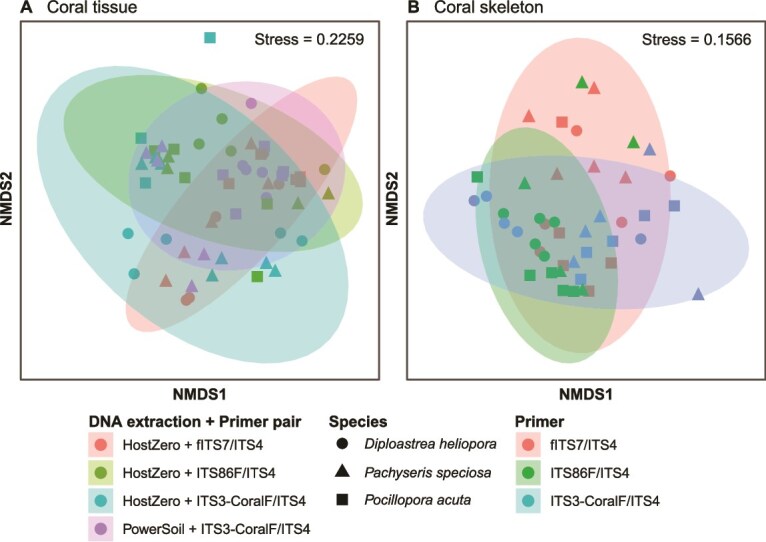
Non-metric multidimensional scaling (NMDS) plot with Bray–Curtis dissimilarities of fungal communities associated with (A) coral tissue and (B) coral skeleton, with different library construction methods. All coral skeleton samples were extracted with the PowerSoil kit.

The NMDS with Bray–Curtis dissimilarities exhibited large overlaps among fungal communities across all library preparation methods (Fig. 6A). Although PERMANOVA highlighted that tissue-associated fungal communities were significantly affected by library construction method (*P* = .001; Table S12), pairwise comparisons revealed differences between communities extracted with PowerSoil paired with ITS3-CoralF/ITS4, and HostZERO with ITS86F/ITS4 and fITS7/ITS4 (Table S14). Fungal communities amplified with ITS3-CoralF/ITS4 were similar across the two extraction methods, and communities extracted with HostZERO were similar across all three primer pairs. This suggests that differences in fungal compositions were driven by the combined effects of extraction method and primer choice, rather than by either factor alone. Fungal communities across library construction methods also had homogenous group dispersions (Table S13). The NMDS plot and PERMANOVA with Jaccard index highlighted similar patterns, with large overlaps across fungal communities across library construction methods (Fig. S4). Of the 214 fungal families observed, 64 were unique to one method, while the 69 families that were shared across comprised 97.16% ± 0.01% across all samples, and were unaffected by the library construction methods (*P* = .0561; Table S15 and Fig. S5).

### Thorough pre-processing of coral skeleton reduced host co-amplification for fungal community characterisation

The ITS3-CoralF primer significantly improved fungal reads recovery on coral skeleton samples extracted with the PowerSoil kit across all coral species (*P* = .002321; Table S8), increasing from an average of 20.10% ± 3.37% with the universal primers to 50.20% ± 8.70% (Fig. 4B). Overall, rarefaction curves highlight that all skeleton samples had sufficient sequencing depth for richness saturation, with a minimum read count of 532 used as the rarefaction threshold (Fig. 5B).

However, ITS3-CoralF yielded communities with lower Shannon diversity of 1.49 ± 0.19 compared to that by fITS7/ITS4 at 2.40 ± 0.22, and lower evenness at 0.386 ± 0.046 compared to the universal primer pairs at 0.610 ± 0.037 (*P* < .05; Tables S9 and S16). Fungal richness was not affected by primer selection, with an average of 56.13 ± 5.95 (Table S17).

The NMDS ordination did not show clear separation across fungal communities by primer sets, although PERMANOVA indicated significant compositional shifts (*P* = .001, Fig. 6B, Table S13), with ITS3-CoralF/ITS4 communities differing significantly from those amplified with fITS7/ITS4 and ITS86F/ITS4 (Table S14). Dispersion analysis confirmed that differences were not driven by heterogeneous group dispersions (*P* = .1216, Table S14). Consistent patterns were observed in the NMDS ordination and PERMANOVA based on the Jaccard index, with fungal communities showing broad overlap across different primers (Fig. S4). Of the 138 identified families, 29 were unique to a single primer pair, while 75 were shared across all three primer pairs. The relative abundance of these shared families were similar between communities amplified with fITS7/ITS4 and ITS3-CoralF/ITS4 at 97.08 ± 0.01%, but was significantly higher than with ITS86F/ITS4 at 85.43 ± 0.04% (*P* = .0007, Table S15).

## Discussion

Characterizing coral-associated fungal communities with the standard metabarcoding workflow of total extracted DNA with universal fungal primer pairs has long been challenged by extensive cross-amplification of coral host DNA, resulting in inadequate fungal sequencing coverage [24, 29, 35]. Likewise, we faced overwhelming coral host co-amplification across all coral tissues samples that were extracted with PowerSoil kit then amplified with the routinely used fITS7/ITS4 and ITS86F/ITS4 primer pairs, generating on average only 0.047% or 15 fungal reads across the two primer pairs. This severely obscured the estimation of fungal diversity associated with corals, and prevented any further compositional analyses and ecological inferences [27].

To tackle this challenge, we evaluated two strategies: selective DNA lysis with HostZERO Microbial DNA kits and a newly designed mismatch primer, ITS3-CoralF. Both approaches substantially alleviated host contamination and enabled robust fungal community profiling across coral species. Fungal read proportions increased to averages of 26.33% with the HostZERO kits, and 75.13% with ITS3-CoralF, transforming datasets that were previously unusable into those suitable for fungal diversity analyses. By directly overcoming host co-amplification, these methods resolve a long-standing barrier to studying coral-associated fungi. Here, we provide recommendations for coral-associated fungi characterisation and hope to catalyse global efforts in understanding their diversity, roles, and ecology.

Both in-silico and in-vitro PCR provide strong evidence that the ITS3-CoralF/ITS4 primer set offers a representative characterisation of marine fungal communities. It maintains broad coverage across fungal sequences, particularly dikaryotic fungi, which typically comprise up to 80% of coral-associated fungal communities [36, 40] and the majority of marine fungi [64]. Alongside fITS7 and ITS86F, ITS3-CoralF also successfully recovered all 10 species in the mock community, indicating their broad taxonomic coverage and resolution within dikaryotic fungi. Importantly, only three differentially abundant orders were identified relative to ITS86F/ITS4 in sediment communities, compared with 10 for fITS7/ITS4. This indicates that primer-specific biases are inherent even across commonly used primers, and that the host-targeting modifications of ITS3-CoralF do not come at a marked expense of capturing a reduced marine mycobiome.

Crucially, incorporating primer-coral mismatches at the 3′ end of the ITS3-CoralF primer effectively reduced host co-amplification from total extracted coral tissue DNA. This modification, on average, yielded an 800-fold increase in fungal reads compared to universal fungal primers, enabling all samples across all three coral species to reach rarefaction without compromising fungal diversity capture. By effectively balancing reduced host co-amplification with broad fungal coverage, the ITS3-CoralF offers a cost-effective strategy that can be readily incorporated into existing metabarcoding workflows for the thorough characterisation of fungal communities associated across corals species. Its lack of amplification to nine other Anthozoan orders further suggests that ITS3-CoralF could be scaled to characterize fungal communities associated with other Anthozoan hosts, which likely face similar host co-amplification challenges when using universal fungal primers.

The host depletion HostZERO kit provide a reliable method for reducing host co-amplification and significantly increasing fungal read recovery. Compared to the typical metabarcoding workflow, the HostZERO kit alone consistently improved fungal reads coverage by 400–1500-fold across the three tested coral species here, which was sufficient for all downstream analyses. Its selective lysis method also enhanced fungal species recovery in sediment samples by up to twofold when paired with universal fungal primers, likely due to the removal of off-target non-fungal eukaryotic DNA that otherwise masks low-abundance fungi [65, 66]. While the effectiveness of the HostZERO kit has been previously demonstrated to enrich bacterial signals [37, 67], our findings further underscore its broader applicability for mitigating host contamination to enrich fungal signals in complex microbiomes.

As such, use of either the ITS3-CoralF primer or HostZERO kit enabled comprehensive characterisation of fungal communities associated with coral tissues. Across library construction method, alpha-diversity indices did not differ drastically, and observed richness was consistently high across all sampled coral individuals [40, 42]. Despite slight compositional biases, all four library construction methods consistently recovered a similar core community of 69 fungal families comprising 97% of tissue-associated communities, and primarily differed in detecting low-abundance taxa. Overall, given its consistent reduction of host co-amplification across the three coral species, we recommend the HostZERO kit paired with universal primers to characterize coral tissue mycobiome communities without significant biases in compositions. However, its extraction cost is approximately triple that of the PowerSoil kit, potentially limiting its adoption in large-scale or resource-constrained studies. As a cost-effective alternative, we suggest using ITS3-CoralF/ITS4 primers on total extracted DNA, which were equally effective compared to the HostZERO kit in reducing host co-amplification, particularly for researchers who already have extracted DNA to study coral-associated bacteria. Given the need to understand holobiont dynamics under climate change [68, 69], the adoption of ITS3-CoralF/ITS4 may offer a more practical path forward for expanding global insights into the diversity and function of fungi associated with corals by directly addressing a major bottleneck in coral mycobiome studies. This primer pair could also be applied to other benthic marine organisms such as sponges, where progress is similarly plagued by host co-amplification [70].

Coral skeletons offer a stable habitat for endolithic fungi, which may play key roles in coral and reef functioning [29]. It is therefore important to characterize fungal communities within the skeletons separately from other compartments to better understand the diversity and compartmentalization of coral-associated fungi [71]. Our results demonstrate that the removal of coral tissue from the skeletons can sufficiently reduce host DNA co-amplification in skeleton samples, enabling adequate recovery of fungal reads even with universal fungal primers, although the ITS3-CoralF/ITS4 primer pair can further increase the number of fungal reads returned.

It is important to recognise that ITS3-CoralF primer may prevent the detection of certain fungal taxa, especially non-dikaryotic fungi, leading to compositional shifts relative to universal primer pairs. Introducing mismatches to reduce coral host amplification came at the cost of creating bias against certain fungal phyla such as Aphelidiomycota and Rozellomycota, which are known to inhabit marine ecosystems and hosts [72, 73]. Despite these biases, ITS3-CoralF/ITS4 was the only primer set in this study that consistently overcame the pervasive challenge of host DNA contamination, increasing fungal reads to levels comparable to well-established host depletion methods to reliably capture fungal diversity across coral species when paired with total extracted DNA. Ultimately, no two primer pairs, even those targeting the same region, yield the same fungal community since none of them cover all fungal groups effectively without biases [43, 74]. With nearly half of all observed fungal families unique to a single primer pair, we recommend the use of multiple primer pairs for a comprehensive characterisation of the mycobiome [75].

Moreover, the improvement of fungal reads coverage did not show strong species-specific effects, improving to at least 40% in *P. speciosa*, compared to 97% in *Pocillopora acuta* and 88% in *D. heliopora*. Slight differences may reflect variations in rDNA copy number [76], or the presence of divergent rDNA pseudogenes with reduced mismatch penalties, as reported in *Acropora* [77]. Although reads were still sufficient across all coral samples for species saturation, increasing microbial reads can facilitate the discovery of low abundance taxa [78]. In-silico analyses also indicate that only Acroporidae corals share two mismatches with ITS3-CoralF which may reduce the effectiveness of ITS3-CoralF. We recommend in-silico primer testing against target host sequences prior to library construction. Annealing specificity may be further enhanced by touchdown PCR [79], and read depth increased by multiplexing fewer samples per run.

Substantial compositional differences were observed between sediment samples extracted with HostZERO and those with PowerSoil kit, with 19 fungal orders showing differential abundance. These shifts were likely driven by differences in fungal susceptibility to the selective lysis stage, which also altered bacterial and viral compositions [67, 80]. Although the major fungal cell wall building blocks are conserved, minor components, including their interactions and dynamics, can vary considerably even within the same genus [81, 82], causing certain taxa to be lysed accidentally alongside host cells during selective lysis stage. In addition, certain pathogenic fungi from Cryptomycota and Blastocladiomycota that reside within host cells [83] and zoospores produced by chytrids all lack typical chitinous cell walls [84], and may therefore be especially vulnerable. Since HostZERO targets intact cells, some recovered compositional biases may also stem from the removal of relic and extracellular DNA during the DNA depletion process, which can make up to 80% of the DNA found in sediments [85, 86]. Nonetheless, the removal of relic DNA can enhance the resolution of ecological patterns and microbial network analyses [87, 88]. Overall, we argue that HostZERO’s ability to uncover rare and low-abundance fungi outweigh the compositional shifts, and recommend its use across all sample types. In light of the vast, unexplored diversity of marine fungi, it is pivotal that we thoroughly characterize fungal diversity to describe the true magnitude of the marine mycobiome [89].

## Conclusion

In coral holobionts, the dominance of host DNA remains a major barrier to effective fungal community profiling. Here, we outline practical and validated recommendations that, despite minor biases in diversity and composition, consistently yield sufficient fungal coverage for robust downstream analyses. Across sample types, we recommend the HostZERO kit paired with universal fungal primers for effective depletion of off-target DNA to improve fungal recovery. For coral tissues, the ITS3-CoralF/ITS4 primer pair offers a cost-effective alternative, suppressing host amplification even from total extracted DNA across the three coral species in this study. For skeleton samples, thorough tissue removal prior to extraction is sufficient to reduce host amplification, though ITS3-CoralF/ITS4 can further enhance fungal detection. Together, these approaches provide a practical and scalable framework that overcomes a central technical barrier in coral mycobiome research, enabling robust, comparable, and reproducible characterisation of fungal communities across coral hosts.

## Supplementary Material

ycag060_Supplemental_File

## Data Availability

The dataset associated with this work is available in the National Centre for Biotechnology Information (NCBI) repository, under BioProject ID: PRJNA1307061 and PRJNA1418264. The R code used for the analyses has been deposited at https://github.com/mingshengg/coral-fungi-characterization.
