# Supplementary material for: Improved characterisation of coral-associated fungal communities using host DNA depletion and a novel ITS primer
Source: ISME Commun. 2026 Mar 13;6(1):ycag060. doi: 10.1093/ismeco/ycag060 (PMC13077293; doi:10.1093/ismeco/ycag060)
Supplement: ycag060_Supplemental_File [file ycag060_supplemental_file.docx]

**1. Supplementary Methods**

**Primer design**

To design the primer, we first downloaded coral and fungal 5.8S sequences from the NCBI database, and aligned them on Geneious Prime (v2024.0.3). For corals, common coral species found in Singapore and other Indo-Pacific reefs such as *Merulina, Porites, Acropora,* and *Echinopora*, and the three species of corals used in this study, were downloaded. ITS sequence for *Pachyseris speciosa* was not available on NCBI and thus was amplified with ITS1F/ITS4 and sequenced with Sanger Sequencing (uploaded onto GenBank under Accession number PX057261.1). For fungi, we looked at existing literature on fungi commonly cultured from corals, and further included other common marine fungi such as *Malessezia* and fungi from Chytridiomycota. A total of 20 coral and 222 fungal sequences, spanning across Ascomycota, Basidiomycota, Chytridiomycota, and Mucoromycota, were downloaded and aligned.

After sequences have been downloaded, we tested commonly used ITS2 forward primers that attach to the 5.8S region to inspect their specificity to coral and fungi DNA. Unsurprisingly, all tested forward primers fITS7, gITS7ngs, ITS3-Mix, and ITS86F had 0 or 1 mismatch with coral sequences. However, we realized that ITS3 sits on the edge of a more variable region in the 5.8S, where shifting the ITS3 primer four bases towards the 3’ end would introduce one to four 3’ mismatches to coral host DNA.

To ensure that the primer design is robust, we followed guidelines from Abd-Elsalam (2003). Namely, the primer has a GC% between 45% and 60% at 47%, with melting temperature kept below 65 °C, and does not contain regions that would allow the primer to anneal to itself to form dimers or secondary structures. The annealing temperature is also very close to that of ITS4 at 62 °C. ITS3-CoralF, however, does not have a GC clamp, or a 3’-end sequence with G’s or C’s, to further increase binding specificity and reduce spurious secondary bands. While ITS3-CoralF has three repeating A’s on the 3’ end which may reduce primer binding efficiency and specificity, this is also observed in another fungal universal forward primer ITS3_KYO2 (5’- GAT GAA GAA CGY AGY RAA -3’), and other eukaryotic universal primers such as LR9 (5’- AGA GCA CTG GGC AGA AA -3’) and NemF-18Sr2b (5’- GGG AAG TAT GGT TGC AAA -3’). On the other hand, fITS7 has an annealing temperature of 55 – 57 °C, and contains a GC clamp, while ITS86F has an annealing temperature of 57 °C, and does not contain a GC clamp.

**DNA extraction with the Zymo HostZERO kits**

Due to centrifugation of the samples during preservation, coral tissue samples were pelleted at the falcon tubes. Approximately 0.01 g of coral tissue pellets was resuspended in 1 mL of autoclaved phosphate buffered saline (PBS) by running through syringe and needle repeatedly for 10 times and gently vortexed for 3 mins. Once tissues have been thoroughly resuspended, 200 µl of sample is added to a fresh 1 ml Host DNA Depletion Solution. The manufacturer’s protocol was followed with the exception of these modifications: During Host DNA Depletion, the samples are rotated for 30 min using end-over-end rotation at room temperature to maximize lysis of host cells. Proteinase K incubation was also increased to 20 min at 55 °C. For the bead beating step of the microbial DNA isolation, samples were processed at speed 8 for 5 min in a Vortex Genie 2 (Scientific Industries, Inc., NY, USA).

The above protocol was also used for sediment samples with the exception that approximately 0.01g of sediment was directly placed in 1 ml Host DNA Depletion Solution for the first step.

**DNA extraction with the PowerSoil PowerLyzer kit**

The same PowerSoil PowerLyzer Kit protocol was applied across all sample types, where the manufacturer’s protocol was followed with the following modifications: For coral skeleton and sediment samples, approximately 0.25g of sample was added into the PowerBead Tube as the first step. On the other hand, approximately 0.25 g of pelleted coral tissue was added directly into the PowerBead Tube. After adding Solution C1, samples were incubated in a water bath at 70 °C for 10 min, then attached to a Vortex Genie 2 for bead beating at speed 8.5 for 10 min. At the final elution step, 50 µL of 10 mM Tris was preheated to 55 °C before adding to the spin column, and incubated for 5 min at 55 °C before centrifugation.

**PCR cycling conditions**

For all PCR reactions, each reaction comprised 12.5 µl of Q5 High-Fidelity 2X Master Mix (New England Biolabs, MA, USA), 1 µl each of 10 µM forward and reverse primer, 2 µl of template DNA, and nuclease-free water to 25 µl. All primers were modified to include Illumina adaptors and overhang sequences. The first round PCR cycling conditions began with an initial denaturation step at 98 °C for 30 s, followed by 35 cycles of 98 °C for 10 s, 63 °C for fITS7 and ITS3-CoralF, or 65 °C for ITS86F for 30 s, and 72°C for 20 s, ending with a final elongation step at 72°C for 2 min. The second multiplexing PCR cycling conditions began with an initial denaturation step at 98°C for 30 s, followed by 8 cycles of 98 °C for 10 s, 56°C for 30 s for all three primer combinations, and 72°C for 20 s, ending with a final elongation step at 72°C for 2 min. All PCR products were cleaned with magnetic beads (Mag-Bind TotalPure NGS, Omega Bio-Tek, USA) at 1:0.8 DNA-to-beads ratio following the Illumina Fungal Metagenomic Sequencing Demonstrated Protocol (<https://sapac.support.illumina.com/downloads/fungal-metagenomic-sequencing-demonstrated-protocol-1000000064940.html>) and visualized on a 1% TAE buffer agarose gel to confirm amplification. Final DNA concentrations of multiplexed PCR products were quantified and normalized before pooling for sequencing.


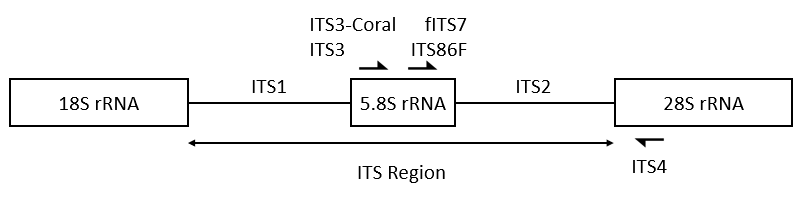


**Figure S1** Schematic map of ribosomal and internal transcribed spacer (ITS) nuclear regions indicating the primers used and/or discussed in this study. The ITS3-CoralF primer is a modified version of ITS3, shifted four bases towards the 3’ end, while ITS86F overlaps fITS7 with two extra bases on the 3’ end, and a degenerate base in the middle.

In previous testing, PCR was also conducted with two other primer pairs, ITS1F/ITS2 (White et al., 1990) and ITS5/5.8S_Fungi (Epp et al., 2012) with the same cycling conditions as above, with annealing temperatures at 51 °C and 58 °C respectively. These primers were used because they were used in other coral-fungi work (Longley et al., 2024; Rabbani et al., 2021; Staley et al., 2017), but in our preliminary analyses less than 1% of sequencing reads were assigned to fungi. The most commonly used universal fungal primer ITS1F/ITS2, in particular, generated only an average of 0.79 % ± 0.33 % fungal reads, with three samples having zero fungal reads. Results are therefore not further reported here.

**Table S1** Percentage of fungal sequences amplified by each forward primer across different fungal phyla from the in-silico PCR.

| Fungal phyla | ITS3-CoralF | fITS7 | ITS86F |
| --- | --- | --- | --- |
| Ascomycota | 94.99 | 96.74 | 96.28 |
| Basidiomycota | 79.83 | 91.03 | 91.08 |
| Chytridiomycota | 85.34 | 94.76 | 94.65 |
| Mucoromycota | 37.01 | 88.3 | 88.2 |
| Blastocladiomycota | 69.62 | 26.58 | 26.58 |
| Aphelidiomycota | 66.67 | 100 | 100 |
| Mortierellomycota | 96.11 | 96.41 | 96.41 |
| Zoopagomycota | 0.17 | 21.97 | 16.44 |
| Olpidiomycota | 98.08 | 98.08 | 98.08 |
| Monoblepharomycota | 88.57 | 91.43 | 91.43 |
| Basidiobolomycota | 0 | 97.06 | 97.06 |
| Kickxellomycota | 0 | 91.41 | 91.41 |
| Entorrhizomycota | 95.45 | 97.73 | 97.73 |
| Neocallimastigomycota | 89.66 | 4.6 | 82.76 |
| Calcarisporiellomycota | 100 | 100 | 100 |
| Sanchytriomycota | 0 | 0 | 0 |
| Entomophthoromycota | 37.5 | 87.5 | 87.5 |
| Glomeromycota | 29.59 | 96.17 | 96.54 |
| Rozellomycota | 24.3 | 76.06 | 54.97 |
| GS01_phy_Incertae_sedis | 0 | 14.79 | 0 |
| Fungi_phy_Incertae_sedis | 70.53 | 79.99 | 79.13 |

**Table S2** Percentage of Anthozoan sequences amplified by each forward primer across different Anthozoan phyla from the in-silico PCR. The newly designed ITS3-CoralF primer did not amplify any Anthozoan sequences from the UNITE database.

| Anthozoa Orders | fITS7 | ITS86F |
| --- | --- | --- |
| Scleractinia | 76.61 | 76.35 |
| Helioporacea | 100 | 100 |
| Actiniaria | 100 | 100 |
| Zoantharia | 97.20 | 97.20 |
| Alcyonacea | 100 | 97.87 |
| Pennatulacea | 100 | 100 |
| Spirularia | 100 | 100 |
| Antipatharia | 100 | 100 |
| Corallimorpharia | 100 | 100 |
| Ceriantharia | 100 | 100 |
|  |  |  |


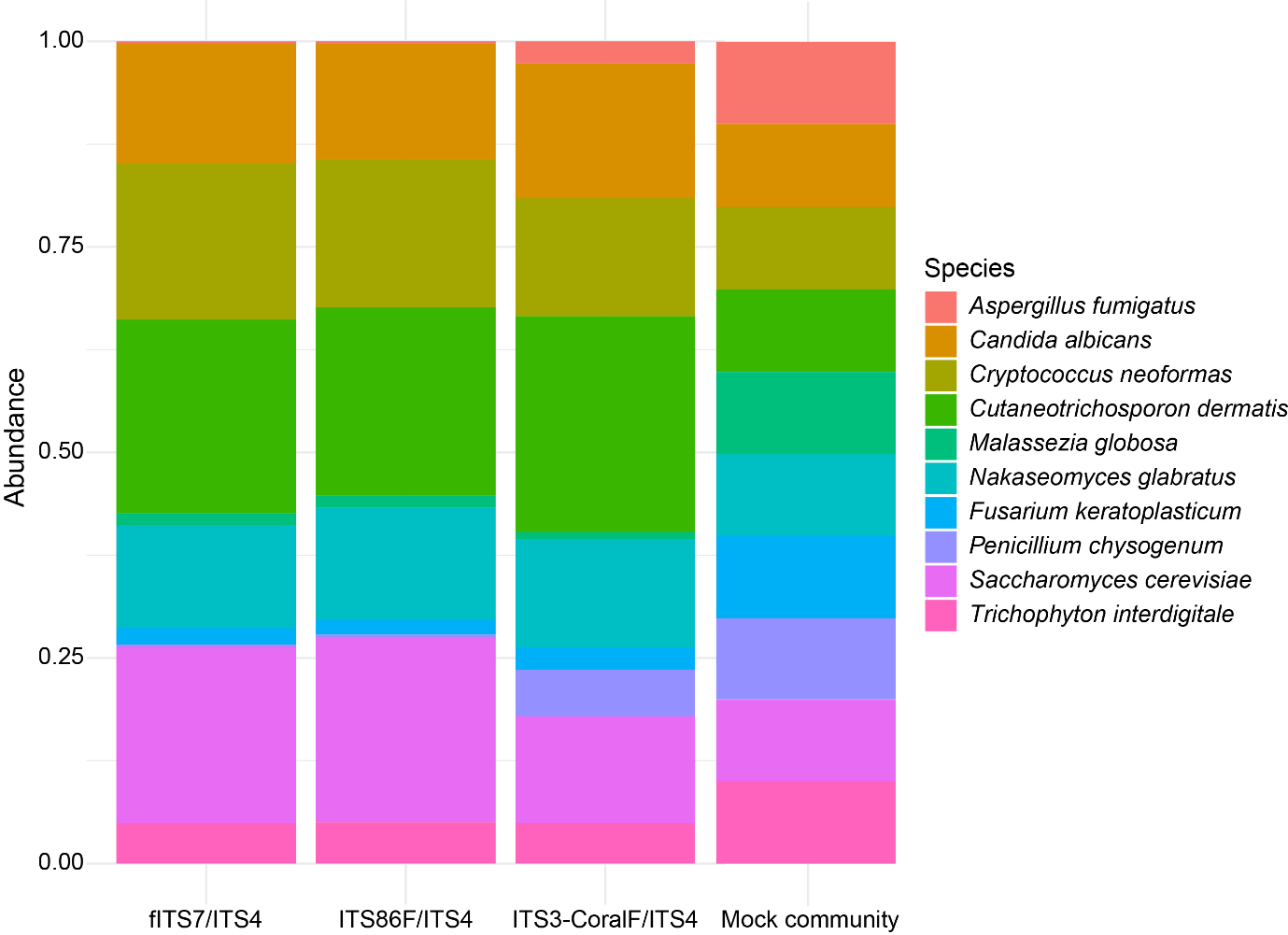


**Figure S2** Composition of fungal species amplified and identified by each primer pair from the Mycobiome Genomic DNA Mix (ATCC – MSA-1010), compared to the actual mock community (10% relative abundance for each species).

**Table S3** Shannon diversity, richness, and evenness of reef sediment fungal communities analyzed with across two DNA extraction kits and three primer pairs.

|  | DNeasy PowerLyzer PowerSoil Kit | | | HostZERO Microbial DNA Kit | | |
| --- | --- | --- | --- | --- | --- | --- |
|  | Shannon | Richness | Evenness | Shannon | Richness | Evenness |
| fITS7/ITS4 | 2.94 ± 0.08 | 65.1 ± 10.8 | 0.733 ± 0.025 | 3.85 ± 0.33 | 199.0 ± 25.9 | 0.741 ± 0.060 |
| ITS86F/ITS4 | 3.76 ± 0.14 | 102.0 ± 23.7 | 0.842 ± 0.017 | 3.54 ± 0.27 | 240.0 ± 30.4 | 0.654 ± 0.046 |
| ITS3-CoralF/ITS4 | 2.82 ± 0.28 | 70.7 ± 24.2 | 0.707 ± 0.035 | 2.82 ± 0.28 | 184.0 ± 37.4 | 0.707 ± 0.035 |

**Table S4** Permutational analysis of variance was conducted to investigate if library construction method significantly affected the composition of fungal communities associated with reef sediments. Low R^2^ values reflect the high complexity and heterogeneity of environmental fungal communities associated with sediments.

|  | Df | SumOfSqs | R^2^ | F | *p-*value |
| --- | --- | --- | --- | --- | --- |
| DNA extraction | 1 | 1.539 | 0.0662 | 3.962 | 0.001 |
| Primer | 2 | 0.987 | 0.0425 | 1.271 | 0.033 |
| Site | 2 | 1.729 | 0.0744 | 2.225 | 0.001 |
| DNA extraction: Primer | 2 | 1.127 | 0.0484 | 1.451 | 0.003 |
| Residual | 46 | 17.866 | 0.7685 |  |  |
| Total | 53 | 23.247 | 1.0000 |  |  |

**Table S5** Permutation test with 999 permutations to assess homogeneity of dispersion across primer and DNA extraction methods.

|  |  | Df | Sum Sq | Mean Sq | F | *p-*value |
| --- | --- | --- | --- | --- | --- | --- |
| Primer | Groups | 2 | 0.000 | 0.000 | 0.358 | 0.7008 |
|  | Residual | 39 | 0.029 | 0.001 |  |  |
| DNA extraction | Groups | 1 | 0.016 | 0.0160 | 9.080 | 0.0040 |
|  | Residual | 40 | 0.092 | 0.0018 |  |  |

**Table S6** Results of pairwise analyses testing to identify pairs with significantly different fungal community compositions associated with reef sediments.

| Pairs | Sum Sq | F Model | R^2^ | *p-*value | Adj *p* |
| --- | --- | --- | --- | --- | --- |
| fITS7/ITS4 + PowerSoil vs ITS86F/ITS4 + PowerSoil | 0.7290 | 1.7882 | 0.1005 | 0.001 | 0.015 |
| fITS7/ITS4 + PowerSoil vs ITS3-CoralF/ITS4 + PowerSoil | 0.7069 | 1.6872 | 0.0954 | 0.001 | 0.015 |
| fITS7/ITS4 + PowerSoil vs fITS7/ITS4 + HostZERO | 0.8654 | 2.1343 | 0.1177 | 0.001 | 0.015 |
| fITS7/ITS4 + PowerSoil vs ITS86F/ITS4 + HostZERO | 0.9257 | 2.3015 | 0.1258 | 0.002 | 0.030 |
| fITS7/ITS4 + PowerSoil vs ITS3-CoralF/ITS4 + HostZERO | 0.8933 | 2.1969 | 0.1207 | 0.001 | 0.015 |
| ITS86F/ITS4 + PowerSoil vs ITS3-CoralF/ITS4 + PowerSoil | 0.7452 | 1.7807 | 0.1001 | 0.001 | 0.015 |
| ITS86F/ITS4 + PowerSoil vs fITS7/ITS4 + HostZERO | 0.7179 | 1.7725 | 0.0997 | 0.002 | 0.030 |
| ITS86F/ITS4 + PowerSoil vs ITS86F/ITS4 + HostZERO | 0.9656 | 2.4036 | 0.1306 | 0.002 | 0.030 |
| ITS86F/ITS4 + PowerSoil vs ITS3-CoralF/ITS4 + HostZERO | 0.9122 | 2.2461 | 0.1231 | 0.002 | 0.030 |
| ITS3-CoralF/ITS4 + PowerSoil vs fITS7/ITS4 + HostZERO | 0.8069 | 1.9383 | 0.1081 | 0.001 | 0.015 |
| ITS3-CoralF/ITS4 + PowerSoil vs ITS86F/ITS4 + HostZERO | 0.8656 | 2.0957 | 0.1158 | 0.001 | 0.015 |
| ITS3-CoralF/ITS4 + PowerSoil vs ITS3-CoralF/ITS4 + HostZERO | 0.8347 | 1.9995 | 0.1111 | 0.001 | 0.015 |
| fITS7/ITS4 + HostZERO vs ITS86F/ITS4 + HostZERO | 0.2973 | 0.7441 | 0.0444 | 0.781 | 1.000 |
| fITS7/ITS4 + HostZERO vs ITS3-CoralF/ITS4 + HostZERO | 0.4088 | 1.0119 | 0.0595 | 0.395 | 1.000 |
| ITS86F/ITS4 + HostZERO vs ITS3-CoralF/ITS4 + HostZERO | 0.2833 | 0.7070 | 0.0423 | 0.923 | 1.000 |


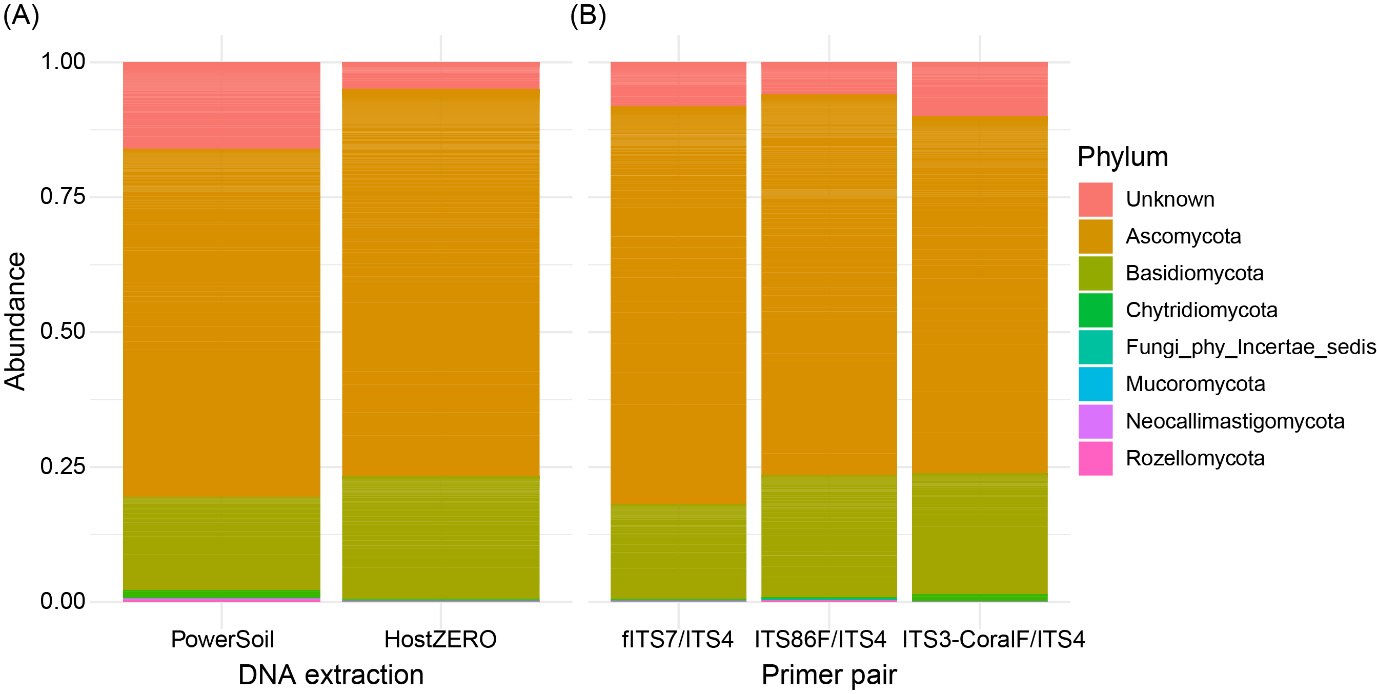


**Figure S3** Compositions of fungal communities with different (A) DNA extraction methods and (B) primer pairs.

**Table S7** Percentage fungal reads across the four main library construction methods, with two DNA extraction kits crossed with two primer pairs (Universal: ITS86F/ITS4 and fITS7/ITS4; New: ITS3-CoralF/ITS4).

|  | *Diploastrea heliopora* | *Pachyseris speciosa* | *Pocillopora acuta* |
| --- | --- | --- | --- |
| PowerSoil + Universal | 0.05 ± 0.02 | 0.05 ± 0.01 | 0.05 ± 0.02 |
| PowerSoil + New | 88.00 ± 3.55 | 40.30 ± 22.8 | 97.10 ± 0.84 |
| HostZERO + Universal | 35.00 ± 9.11 | 22.80 ± 5.92 | 21.80 ± 3.56 |
| HostZERO + New | 75.00 ± 6.97 | 45.60 ± 9.37 | 84.50 ± 9.71 |

**Table S8** ANOVA was conducted to investigated if percentage fungal reads of (A) coral tissue and (B) skeleton samples were significantly affected by primer pair, species and/or DNA extraction, and their interaction effects. Percentage fungal reads were log-transformed to better meet ANOVA assumptions of normality and homoscedasticity.

|  |  | Df | Sum Sq | Mean Sq | F value | *p*-value |
| --- | --- | --- | --- | --- | --- | --- |
| (A)  Tissues | DNA extraction | 1 | 202.34 | 202.34 | 287.00 | 0.0000 |
|  | Primer | 2 | 256.78 | 128.39 | 182.11 | 0.0000 |
|  | Species | 2 | 7.48 | 3.74 | 5.31 | 0.0076 |
|  | DNA extraction: Primer | 2 | 143.37 | 71.69 | 101.68 | 0.0000 |
|  | DNA extraction: Species | 2 | 0.93 | 0.46 | 0.66 | 0.5215 |
|  | Primer: Species | 4 | 5.78 | 1.44 | 2.05 | 0.0989 |
|  | DNA extraction: Primer: Species | 4 | 5.31 | 1.33 | 1.88 | 0.1252 |
|  | Residuals | 60 | 42.30 | 0.71 |  |  |
| (B) Skeletons | Primer | 2 | 11.1808 | 5.5904 | 8.1201 | 0.0012 |
|  | Species | 2 | 11.5459 | 5.7729 | 8.3852 | 0.0010 |
|  | Primer: Species | 4 | 1.9109 | 0.4777 | 0.6939 | 0.6011 |
|  | Residuals | 37 | 24.7848 | 0.6885 |  |  |

**Table S9** ANOVA was conducted on to investigate if library construction method (DNA extraction + Primer choice) or primer choice significantly affected the Shannon diversity, richness, and community evenness of fungal communities associated with the (A) tissues and (B) skeletons of three species of corals. For coral tissues, Shannon diversity and richness indices were log-transformed, while for skeletons, richness index was log-transformed to better meet ANOVA assumptions of normality and homoscedasticity.

|  |  |  | Df | Sum Sq | Mean Sq | F value | *p*-value |
| --- | --- | --- | --- | --- | --- | --- | --- |
| (A) Tissues | Shannon | Method | 3 | 2.903 | 0.967 | 3.211 | 0.0311 |
|  |  | Species | 2 | 1.331 | 0.665 | 2.208 | 0.1209 |
|  |  | Method: Species | 6 | 2.833 | 0.472 | 1.567 | 0.1774 |
|  |  | Residuals | 48 | 14.464 | 0.301 |  |  |
|  | Richness | Method | 3 | 3.421 | 1.140 | 2.387 | 0.0805 |
|  |  | Species | 2 | 0.738 | 0.369 | 0.773 | 0.4671 |
|  |  | Method: Species | 6 | 9.917 | 1.652 | 3.460 | 0.0064 |
|  |  | Residuals | 48 | 22.926 | 0.477 |  |  |
|  | Evenness | Method | 3 | 0.486 | 0.162 | 5.409 | 0.0028 |
|  |  | Species | 2 | 0.067 | 0.033 | 1.119 | 0.3348 |
|  |  | Method: Species | 6 | 0.353 | 0.058 | 1.966 | 0.0892 |
|  |  | Residuals | 48 | 1.437 | 0.029 |  |  |
| (B) Skeleton | Shannon | Primer | 2 | 2.642 | 1.321 | 7.809 | 0.0015 |
|  |  | Species | 2 | 1.553 | 0.776 | 4.590 | 0.0168 |
|  |  | Primer: Species | 4 | 2.384 | 0.596 | 3.523 | 0.0159 |
|  |  | Residuals | 37 | 6.091 | 0.169 |  |  |
|  | Richness | Primer | 2 | 2153 | 1076 | 0.682 | 0.5120 |
|  |  | Species | 2 | 444 | 222 | 0.140 | 0.8692 |
|  |  | Primer: Species | 4 | 10645 | 2661 | 1.686 | 0.1745 |
|  |  | Residuals | 37 | 56814 | 1578 |  |  |
|  | Evenness | Primer | 2 | 0.589 | 0.294 | 10.683 | 0.0002 |
|  |  | Species | 2 | 0.390 | 0.195 | 7.086 | 0.0025 |
|  |  | Primer: Species | 4 | 0.151 | 0.037 | 1.370 | 0.2635 |
|  |  | Residuals | 37 | 0.992 | 0.027 |  |  |

**Table S10** Alpha diversity indices of fungal communities associated with tissue samples across the three coral species, constructed with one of the four different ways.

|  | Shannon | Richness | Evenness |
| --- | --- | --- | --- |
| PowerSoil + ITS3-CoralF/ITS4 | 1.64 ± 0.16 | 52.7 ± 5.7 | 0.456 ± 0.056 |
| HostZERO + fITS7/ITS4 | 2.25 ± 0.12 | 32.5 ± 5.4 | 0.686 ± 0.034 |
| HostZERO + ITS86F/ITS4 | 1.97 ± 0.17 | 56.4 ± 12.8 | 0.586 ± 0.055 |
| HostZERO + ITS3-CoralF/ITS4 | 1.89 ± 0.14 | 63.5 ± 9.2 | 0.490 ± 0.039 |

**Table S11** Results of pairwise analyses testing to identify pairs with significantly different fungal richness associated with coral tissues.

| Species | (A) Contrast | Estimate | *t*-ratio | *p-*value |
| --- | --- | --- | --- | --- |
| *Diploastrea heliopora* | (PowerSoil+ITS3-CoralF/ITS4) - (HostZERO+fITS7/ITS4) | 0.904 | 2.068 | 0.1782 |
|  | (PowerSoil+ITS3-CoralF/ITS4) - (HostZERO+ITS86F/ITS4) | 0.451 | 1.031 | 0.7321 |
|  | (PowerSoil+ITS3-CoralF/ITS4) - (HostZERO+ITS3-CoralF/ITS4) | -0.105 | -0.241 | 0.9950 |
|  | (HostZERO+fITS7/ITS4) - (HostZERO+ITS86F/ITS4) | -0.453 | -1.037 | 0.7287 |
|  | (HostZERO+fITS7/ITS4) - (HostZERO+ITS3-CoralF/ITS4) | -1.009 | -2.309 | 0.1102 |
|  | (HostZERO+ITS86F/ITS4) - (HostZERO+ITS3-CoralF/ITS4) | -0.556 | -1.272 | 0.5849 |
| *Pachyseris speciosa* | (PowerSoil+ITS3-CoralF/ITS4) - (HostZERO+fITS7/ITS4) | -0.133 | -0.305 | 0.9901 |
|  | (PowerSoil+ITS3-CoralF/ITS4) - (HostZERO+ITS86F/ITS4) | -0.882 | -2.180 | 0.1435 |
|  | (PowerSoil+ITS3-CoralF/ITS4) - (HostZERO+ITS3-CoralF/ITS4) | -0.332 | -0.760 | 0.8720 |
|  | (HostZERO+fITS7/ITS4) - (HostZERO+ITS86F/ITS4) | -0.749 | -1.851 | 0.2627 |
|  | (HostZERO+fITS7/ITS4) - (HostZERO+ITS3-CoralF/ITS4) | -0.199 | -0.455 | 0.9683 |
|  | (HostZERO+ITS86F/ITS4) - (HostZERO+ITS3-CoralF/ITS4) | 0.550 | 1.359 | 0.5307 |
| *Pocillopora acuta* | (PowerSoil+ITS3-CoralF/ITS4) - (HostZERO+fITS7/ITS4) | 0.668 | 1.529 | 0.4284 |
|  | (PowerSoil+ITS3-CoralF/ITS4) - (HostZERO+ITS86F/ITS4) | 1.728 | 3.425 | 0.0067 |
|  | (PowerSoil+ITS3-CoralF/ITS4) - (HostZERO+ITS3-CoralF/ITS4) | -0.026 | -0.060 | 0.9999 |
|  | (HostZERO+fITS7/ITS4) - (HostZERO+ITS86F/ITS4) | 1.060 | 2.100 | 0.1677 |
|  | (HostZERO+fITS7/ITS4) - (HostZERO+ITS3-CoralF/ITS4) | -0.695 | -1.589 | 0.3943 |
|  | (HostZERO+ITS86F/ITS4) - (HostZERO+ITS3-CoralF/ITS4) | -1.755 | -3.476 | 0.0058 |

**Table S12** Permutational analysis of variance was conducted to investigate if library construction method, coral species, and their interaction effects, significantly affected the composition of fungal communities associated with (A) coral tissue and (B) coral skeleton samples.

|  |  | Df | SumOfSqs | R^2^ | F | *p-*value |
| --- | --- | --- | --- | --- | --- | --- |
| (A) Tissue | Method | 3 | 1.7486 | 0.07693 | 2.7139 | 0.001 |
|  | Species | 2 | 2.6880 | 0.11826 | 2.7813 | 0.001 |
|  | Method: Species | 6 | 2.8305 | 0.12452 | 1.4643 | 0.024 |
|  | Residual | 48 | 15.4635 | 0.68029 |  |  |
|  | Total | 59 | 22.7306 | 1.00000 |  |  |
| (B) Skeleton | Primer | 2 | 1.2729 | 0.08508 | 2.1952 | 0.002 |
|  | Species | 2 | 1.7352 | 0.11597 | 2.9924 | 0.001 |
|  | Primer: Species | 4 | 1.5165 | 0.10136 | 1.3076 | 0.078 |
|  | Residual | 18 | 10.4376 | 0.69760 |  |  |
|  | Total | 26 | 14.9622 | 1.00000 |  |  |

**Table S13** Permutation test with 999 permutations to assess homogeneity of dispersion across fungal communities with different library construction methods and primer pairs in (A) coral tissues and (B) skeletons.

|  |  | Df | Sum Sq | Mean Sq | F | *p-*value |
| --- | --- | --- | --- | --- | --- | --- |
| (A) Tissues | Groups | 3 | 0.001425 | 0.00047507 | 0.6839 | 0.5656 |
|  | Residual | 56 | 0.0338899 | 0.00069463 |  |  |
| (B) Skeletons | Groups | 2 | 0.0030285 | 0.00151423 | 2.2164 | 0.1216 |
|  | Residual | 42 | 0.0286934 | 0.00068318 |  |  |

**Table S14** Results of pairwise analyses testing to identify pairs with significantly different fungal community compositions associated with (A) coral tissues and (B) skeletons. Coral tissue-associated fungal communities amplified with fITS7/ITS4 and ITS86F/ITS4 were similar, and were thus combined together here as universal primers.

|  | Pairs | Sum Sq | F Model | R^2^ | *p-*value | Adj *p* |
| --- | --- | --- | --- | --- | --- | --- |
| (A) Tissues | HostZERO+Universal vs HostZERO+IT3-CoralF/ITS4 | 1.0385 | 3.0989 | 0.1103 | 0.003 | 0.009 |
|  | HostZERO+Universal vs PowerSoil+IT3-CoralF/ITS4 | 2.0488 | 6.0764 | 0.1955 | 0.001 | 0.003 |
|  | HostZERO+IT3-CoralF/ITS4 vs PowerSoil+IT3-CoralF/ITS4 | 0.4938 | 1.4027 | 0.0806 | 0.183 | 0.549 |
| (B) Skeletons | fITS7/ITS4 vs ITS86F/ITS4 | 0.4290 | 1.4567 | 0.0834 | 0.109 | 0.327 |
|  | fITS7/ITS4 vs ITS3-CoralF/ITS4 | 1.0118 | 3.2591 | 0.1692 | 0.003 | 0.009 |
|  | ITS86F/ITS4 vs ITS3-CoralF/ITS4 | 1.0833 | 3.8325 | 0.1932 | 0.003 | 0.009 |


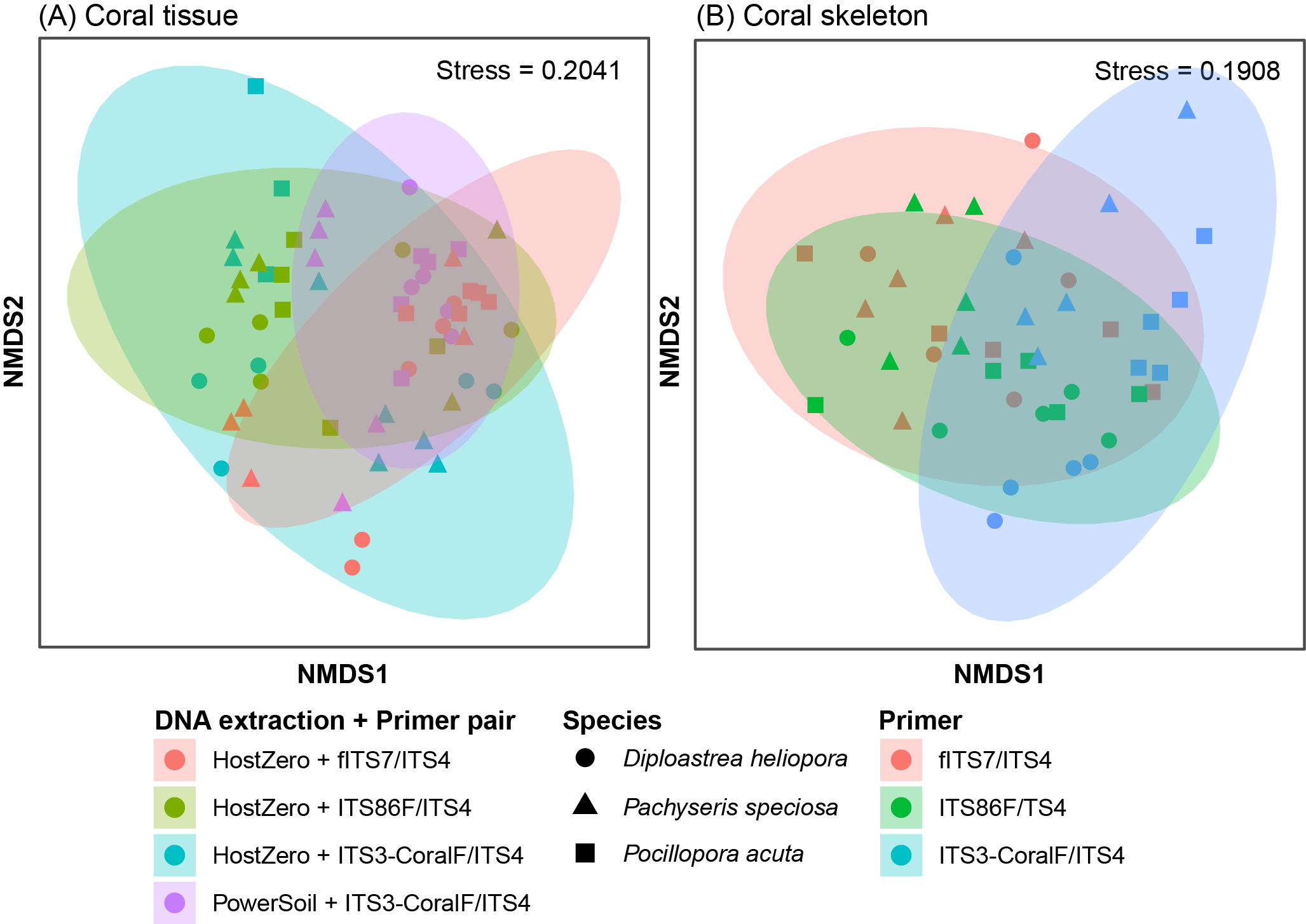


**Figure S4** Non-metric multidimensional scaling (NMDS) plot with Jaccard index of fungal communities associated with (A) coral tissue and (B) coral skeleton, with different library construction methods. All coral skeleton samples were extracted with the PowerSoil kit.


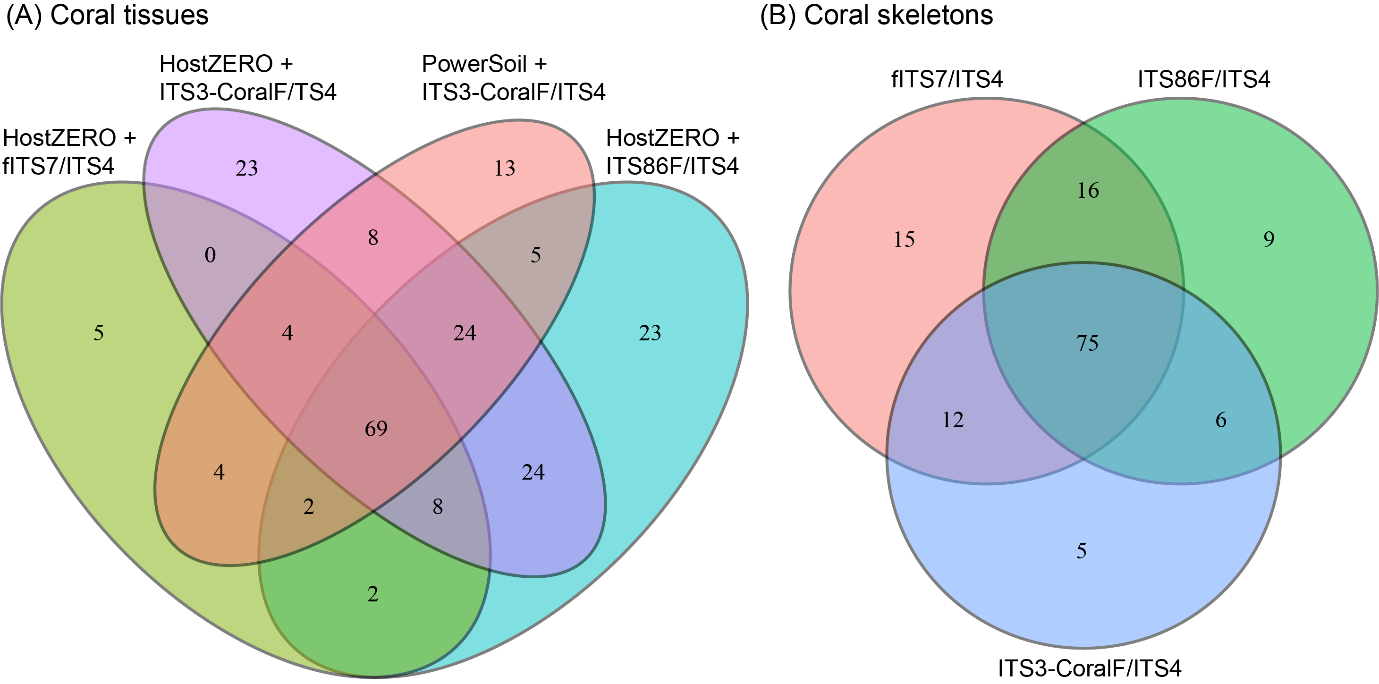


**Figure S5** Fungal families shared between (A) library construction methods (DNA extraction + primer pair) in coral tissues and (B) primer pair in coral skeletons.

**Table S15** ANOVA was used to assess if the relative abundance of the 17 and 19 families shared across the four library construction methods in (A) coral tissues and (B) skeletons, were significantly affected by the library construction methods, coral species, and their interaction effects.

|  |  | Df | Sum Sq | Mean Sq | F value | *p*-value |
| --- | --- | --- | --- | --- | --- | --- |
| (A) Tissues | Method | 3 | 0.0525 | 0.0175 | 3.0806 | 0.0561 |
|  | Species | 2 | 0.0129 | 0.0064 | 1.1328 | 0.3306 |
|  | Method: Species | 6 | 0.0658 | 0.0110 | 1.9322 | 0.0947 |
|  | Residuals | 48 | 0.2727 | 0.0057 |  |  |
| (B) Skeletons | Primer | 2 | 0.6071 | 0.3035 | 9.0165 | 0.0007 |
|  | Species | 2 | 0.0860 | 0.0429 | 1.2771 | 0.2912 |
|  | Primer: Species | 4 | 0.2879 | 0.0720 | 2.1383 | 0.0961 |
|  | Residuals | 36 | 1.2119 | 0.0337 |  |  |

**Table S16** Results of pairwise analyses testing to identify pairs with significantly different fungal richness of fungal communities associated with coral skeletons.

| Index | Contrast | Estimate | *t-*ratio | *p-*value |
| --- | --- | --- | --- | --- |
| Shannon | (fITS7/ITS4) - (ITS86F/ITS4) | 0.2433 | 1.6199 | 0.2504 |
|  | (fITS7/ITS4) - (ITS3-CoralF/ITS4) | 0.5906 | 3.9318 | 0.0010 |
|  | (ITS86F/ITS4) - (ITS3-CoralF/ITS4) | 0.3472 | 2.3119 | 0.0669 |
| Evenness | (fITS7/ITS4) - (ITS86F/ITS4) | 0.1087 | 1.7922 | 0.1866 |
|  | (fITS7/ITS4) - (ITS3-CoralF/ITS4) | 0.2781 | 4.5862 | 0.0002 |
|  | (ITS86F/ITS4) - (ITS3-CoralF/ITS4) | 0.1694 | 2.7940 | 0.0220 |

**Table S17** Alpha diversity indices of fungal communities associated with skeleton samples across the three coral species, constructed with one of the three primer sets.

|  | Shannon | Richness | Evenness |
| --- | --- | --- | --- |
| ITS3-CoralF/ITS4 | 1.49 ± 0.19 | 65.6 ± 8.3 | 0.386 ± 0.046 |
| fITS7/ITS4 | 2.38 ± 0.21 | 52.2 ± 11.0 | 0.661 ± 0.049 |
| ITS86F/ITS4 | 1.91 ± 0.20 | 49.3 ± 10.8 | 0.556 ± 0.053 |

**2. ASV-based analysis**

To account for differences in amplicon length and primer binding sites, ITS2 regions were first extracted from raw reads using ITSxpress2 (Einarsson & Rivers, 2024) with the paired-end merging approach without taxon filtering, generating ITS2 regions from both forward and reverse reads. Amplicon sequence variants (ASVs) were then inferred using DADA2 (Callahan et al., 2016) using only the ITS2 from forward reads to maximise precision in fungal community characterisation (Pauvert et al., 2019).


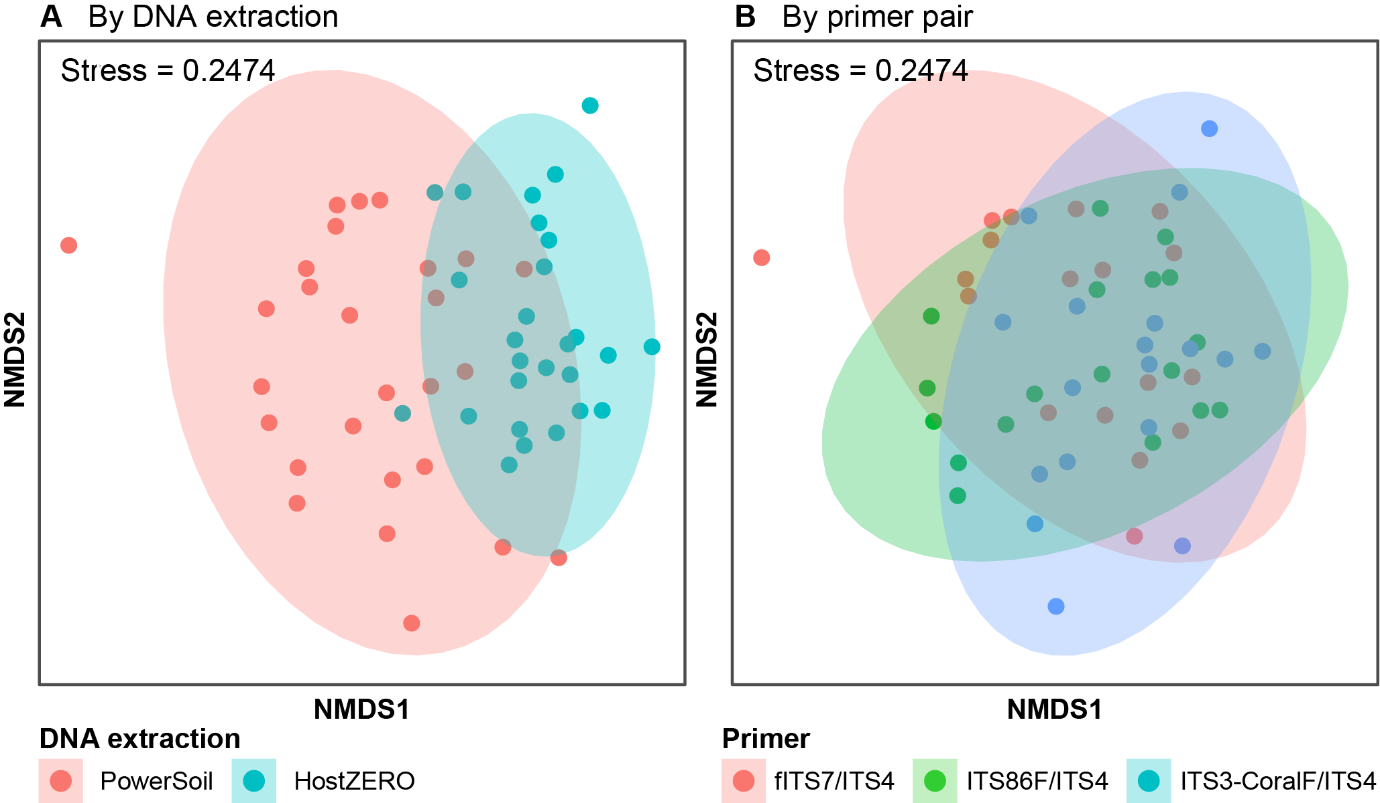


**Figure S6** Non-metric multidimensional scaling (NMDS) plot with Bray-Curtis dissimilarities of fungal communities associated with reef sediments, coloured (A) by DNA extraction and (B) by primer pair.


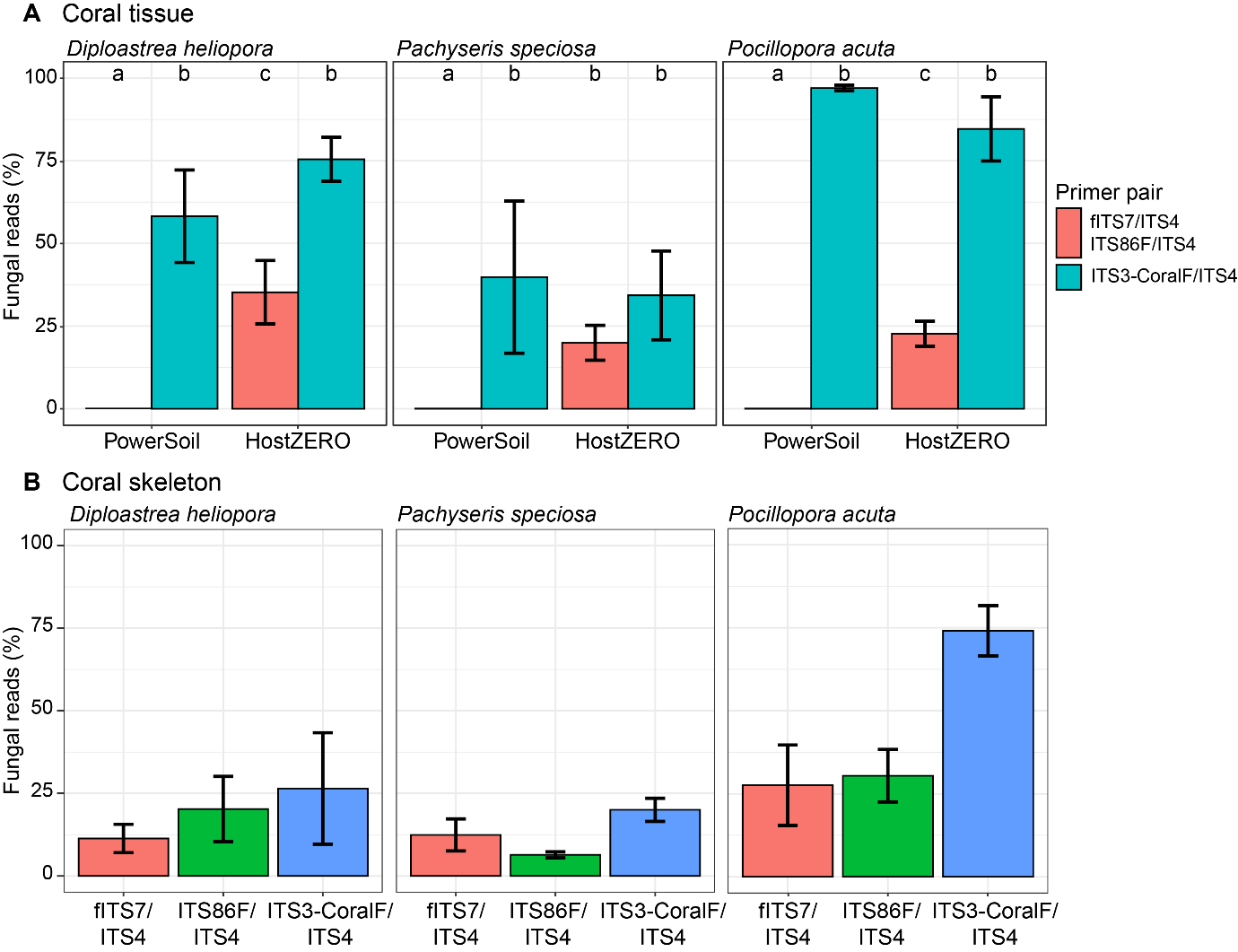


**Figure S7** Differences in percentage fungal reads across different coral species, and DNA extraction methods and/or primer pairs in (A) coral tissue and (B) coral skeleton samples. Letters indicate statistically significant differences within each coral species. Universal fungal primer pairs fITS7/ITS4 and ITS86F/ITS4 are represented together in coral tissue samples for clarity of presentation.


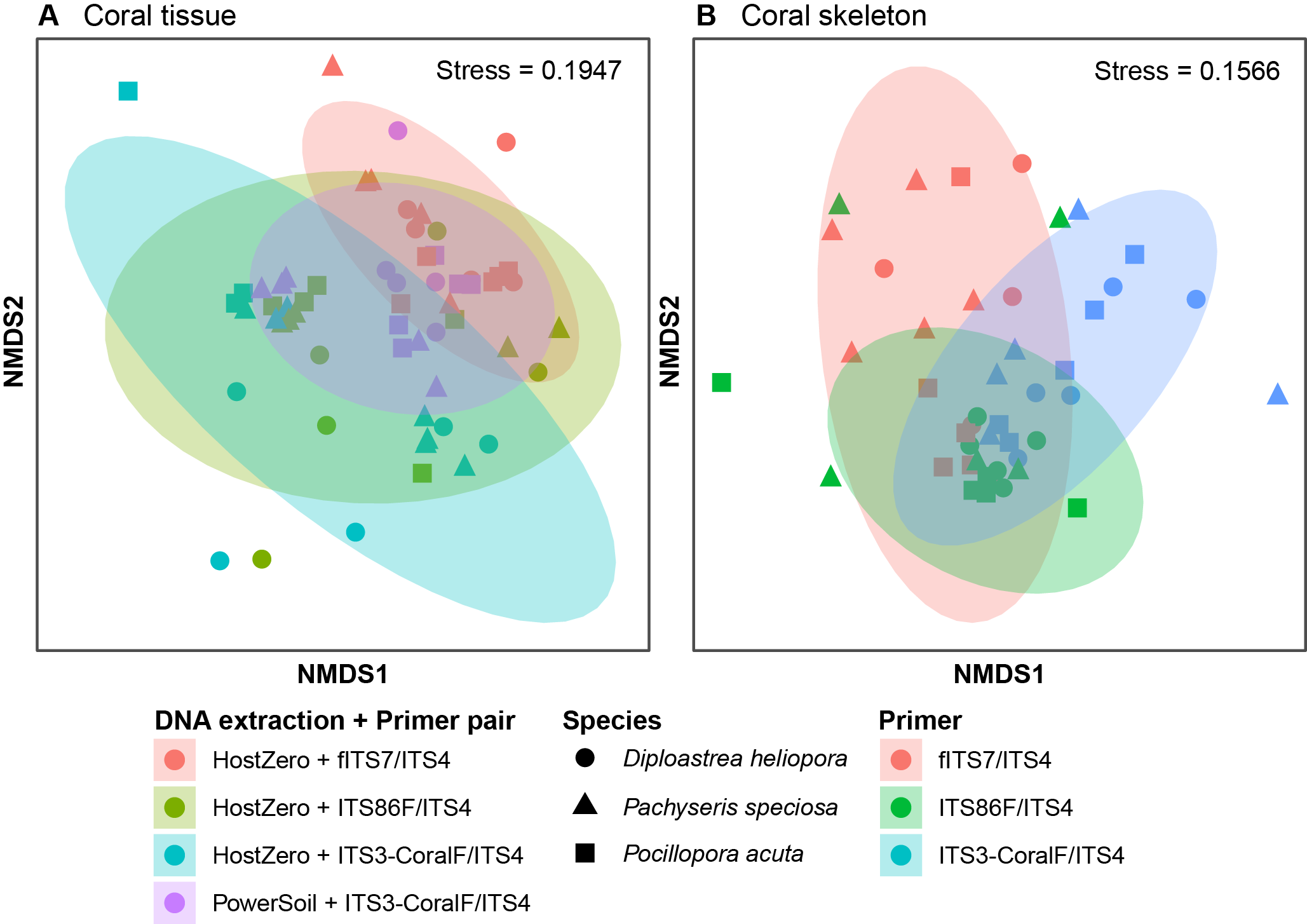


**Figure S8** Non-metric multidimensional scaling (NMDS) plot with Bray-Curtis dissimilarities of fungal communities associated with (A) coral tissue and (B) coral skeleton, with different library construction methods. All coral skeleton samples were extracted with the PowerSoil kit.
